# Targeted Modification of a Novel Amphibian Antimicrobial Peptide from *Phyllomedusa tarsius* to Enhance Its Activity against MRSA and Microbial Biofilm

**DOI:** 10.3389/fmicb.2017.00628

**Published:** 2017-04-19

**Authors:** Yitian Gao, Di Wu, Lei Wang, Chen Lin, Chengbang Ma, Xinping Xi, Mei Zhou, Jinao Duan, Olaf R. P. Bininda-Emonds, Tianbao Chen, Chris Shaw

**Affiliations:** ^1^Natural Drug Discovery Group, School of Pharmacy, Queen's University BelfastBelfast, UK; ^2^College of Basic Medical Science, Zhejiang Chinese Medial UniversityHangzhou, China; ^3^Jiangsu Key Laboratory for Traditional Chinese Medicine Formulae Research, Nanjing University of Chinese MedicineNanjing, China; ^4^AG Systematik und Evolutionsbiologie, IBU—Faculty V, Carl von Ossietzky University OldenburgOldenburg, Germany

**Keywords:** amphibian skin secretion, molecular cloning, medusin, analog design, MRSA, antibiofilm

## Abstract

Antimicrobial peptides (AMPs) in the skin secretions of amphibians are fundamental components of a unique defense system that has evolved to protect these hosts from microbial invasion. Medusins constitute a recently-discovered AMP family from phyllomedusine leaf frog skin and exhibit highly-conserved structural characteristics. Here, we report a novel medusin, medusin-PT, from the skin secretion of the Tarsier Leaf Frog, *Phyllomedusa tarsius*. The mature peptide was initially identified from its cloned biosynthetic precursor-encoding cDNA as obtained by the rapid amplification of cDNA ends (RACE) method. Reverse-phase HPLC and tandem mass spectrometry confirmed both the presence of medusin-PT in the skin secretion and its primary structure. In a range of bioassays, medusin-PT exhibited antimicrobial activity against only the Gram-positive bacterium *Staphylococcus aureus* at 64 μg/ml. However, after directed changes to enhance the cationicity and amphipathicity of the peptide structure, three analog showed more potent antimicrobial activity against several additional bacteria including the antibiotic-resistant bacterium MRSA. In addition, these analog exhibited activity against microbial biofilm (minimum biofilm inhibitory and eradication concentrations of 32 μg/ml and over 64 μg/ml, respectively). These data provide evidence that medusins might be promising candidates as novel antibiotic leads and that the targeted modification of a natural AMP can both improve its efficacy so as to provide new insights into antibiotic design and development.

## Introduction

Antibiotics represent one of the most important discoveries in last century and have addressed the treatment of numerous infectious diseases. For example, the Gram-negative bacteria *Staphylococcus aureus* is a common and important pathogen that, without treatment, can cause a broad spectrum of diseases, such as toxic shock syndrome, lung abscess, sepsis, and endocarditis. It also often forms a strong biofilm and can cause chronic disease (Lowy, 1998). Although antibiotics remain a major therapeutic strategy today, their efficacy in this context has gradually declined because of the increasing development of multi-drug resistant microbes through their indiscriminate and improper overuse (Imperial and Ibana, 2016). Such “superbugs” present an important health risk. For example, methicillin-resistant *S. aureus* (MRSA) can cause skin, soft-tissue, respiratory, bone joint, and endovascular disordered infections and can lead to multiple malignant diseases without effective treatment (Lowy, 1998). Currently, antimicrobial peptides (AMPs) are a major focus of attention as an alternative to conventional antibiotics because of their potent and broad antimicrobial activities, potency against multi-drug resistant bacteria and prevention of biofilm formation (Park et al., 2011). AMPs have also been shown to modulate the host's immune response to neutralize the pro-inflammatory agent lipopolysaccharide and to induce immune cell chemoattraction and cell proliferation (Rosenfeld et al., 2006). Therefore, a focused development of AMPs, including targeted changes to enhance their therapeutic indices and to decrease their treatment risks, could act as an important step to alleviate the pressures on conventional antibiotics.

Skin secretions of anurans in particular are known as among the most abundant sources of natural broad-spectrum AMPs, some of which could have the potential to become therapeutic agents (Giuliani and Rinaldi, 2010). Despite their high structural and sequence heterogeneity, AMPs exhibit some common characteristics, such as comprising 12–100 amino acids, several cationic charges, amphipathicity, and a variety of typical secondary structures (α-helices, β-sheets, and extended and flexible loops) (Porto et al., 2012). These diverse, but, at the same time, conserved structures offer many template-based engineering approaches for developing novel antibiotic candidates. For example, pexiganan, a successful antibiotic candidate in phase III health trials, was modified from magainin, which itself was initially isolated from frog skin secretion (Fjell et al., 2012). In addition, computer-aided molecular modeling based on the physiochemical properties of AMPs has been used to alter the net charges and amphipathicity of natural AMPs so as to increase their therapeutic indices by simultaneously enhancing their bioactivities while decreasing their cytotoxicity (Irazazabal et al., 2016).

In this study, we report on the structural and functional properties of a novel AMP, named medusin-PT, that was isolated from the skin secretion of the Tarsier Leaf Frog, *Phyllomedusa tarsius* using “shotgun” cloning technology and mass spectrometry. In addition to the basic characterisation of medusin-PT, an important aspect of this work is that, through rational structural modification, three analogs of it were designed and synthesized and we were able to show that each displayed higher potencies compared to the natural AMP as well as broader spectra of actions and effectiveness not only against planktonic microorganisms but also against biofilms.

## Materials and methods

### Acquisition of *Phyllomedusa tarsius* skin secretions

The Tarsier Leaf Frog, *P. tarsius*, is a wild Peruvian species whose skin secretions were commercially acquired from Peru Biotech E.I.R.L. These were obtained, in turn, by the combination of gentle electrical transdermal stimulation (<20 s with platinum electrodes; 6 V DC; 4 ms pulse-width; 50 Hz) (Tyler et al., 1992) with hand massaging of the dorsal skin until a pronounced white skin secretion was evident. The foamy white skin secretion was harvested by rinsing from the skin with deionised water and immediately snap frozen in liquid nitrogen. After lyophilisation, samples were stored at −20°C for future analyses.

### Molecular cloning of medusin-PT precursor-encoding cDNA from a secretion-derived cDNA library of *P. tarsius*

Polyadenylated mRNA was extracted from the freeze-dried skin secretion using a Dynabeads® mRNA Direct™ kit (Dynal Biotech, UK). The isolation of polyadenylated mRNA was based on matched-pairing between adenine and thymine bases. The first-strand cDNAs were obtained by reverse transcription and the cDNA libraries were constructed by means of RACE procedures using an Advantage™ 2 PCR Kit (BD Clontech, UK). The sense primer (S1; 5′-ACTTTCYGAWTTRYAAGMCCAAABATG-3′; Y = C+T, W = A+T, R = A+G, M = A+C, B = T+C+G) was designed from a highly-conserved segment of the 5′-untranslated regions of phylloxin cDNA from *Phyllomedusa bicolor* (EMBL accession no. AJ251876) and of the opioid peptide cDNA from *Pachymedusa dacnicolor* (EMBL accession no. AJ005443). The nested universal primer supplied with the kit was used as the antisense primer. RACE products were subjected to purification and cloned using a pGEM®-T Easy Vector System (Promega, UK). An ABI 3100 automated capillary sequencer was used to generate the nucleotide sequences. The EMBL accession numbers of the sequences discovered are as follows: Medusin-PT (LT591889), and Medusin-AC (CCI79382), Medusin-PD (CCI79381) and Medusin-PH (CCI79383) from the frog skins of *Agalychnis callidryas, Pachymedusa dacnicolor*, and *Phyllomedusa hypochondrialis*, respectively (Xi et al., 2013).

### Isolation and structural characterisation of the putative novel cDNA-encoded AMP

An additional 5 mg of lyophilised skin secretion were reconstituted in 1 ml of deionised water containing 0.05% (v/v) trifluoroacetic acid (TFA). The sample was then centrifuged at 5,000 × *g* for 20 min and the supernatant was injected into a reversed-phase HPLC (RP-HPLC) system, followed by elution with a gradient programme from 0.05/99.95 (v/v) TFA/water to 0.05/19.95/80.00 (v/v/v) TFA/water/acetonitrile in 240 min. The column effluent was monitored by UV absorbance at 214 and 280 nm, and fractions were collected automatically at 1 min intervals. Chromatographic fractions were then analyzed by a matrix-assisted, laser desorption, ionization, time-of-flight mass spectrometer (MALDI-TOF MS) (Perspective Biosystems, USA) in positive detection mode using α-cyano-4-hydroxycinnamic acid as the matrix. The system was calibrated with known peptide standards to a precision of ±0.1%. Peptides in fractions having identical molecular masses to that calculated for the peptide deduced from the cloned cDNA were then subjected to primary structural analysis by MS/MS fragmentation sequencing using an LCQ-Fleet electrospray ion-trap mass spectrometer (Thermo Fisher Scientific, USA).

### Peptide synthesis

Both the natural peptide and the analogs designed from it (see below) were chemically synthesized by a standard solid-phase method to obtain sufficient samples for assaying bioactivities. The process employed Rink amide resin and standard Fmoc chemistry and was performed in an automatic PS4 peptide synthesizer (Protein Technologies, USA). After synthesis, all the peptides were cleaved from the resin and deprotected. Their purity was determined by RP-HPLC and MALDI-TOF MS.

### Minimum inhibitory concentration (MIC) and minimum bactericidal concentration (MBC) assays

The antimicrobial activity of each synthetic peptide was evaluated using the broth dilution method (Wiegand et al., 2008). In brief, different concentrations of the peptides were incubated with microorganisms under defined conditions. Six different microorganisms were used in the assay: the Gram-positive bacteria *Staphylococcus aureus* (NCTC10788), *Enterococcus faecalis* (NCTC12697), and methicillin-resistant *Staphylococcus aureus* (MRSA) (NCTC 12493); the Gram-negative bacteria *Escherichia coli* (NCTC 10418) and *Pseudomonas aeruginosa* (ATCC 27853); and the yeast *Candida albicans* (NCYC 1467). Each microorganism was cultured in fresh Mueller-Hinton broth (MHB) overnight and then subcultured in fresh medium to a cell density equivalent to 10^8^ colony-forming units (CFU)/ml. The microbial suspension was diluted with fresh MHB to a concentration of 1 × 10^5^ CFU/ml. Peptide samples were dissolved in the culture medium MHB to prepare a 100-fold stock solutions and then 1 μl of each peptide solution was mixed with 99 μl of diluted bacterial culture to get a final concentration range from 1 to 512 μg/ml in two-fold dilution. After a 16–20 h incubation period, the absorbance values of the wells of the 96-well plates were determined at 550 nm using a Synergy HT plate reader (Biotech, USA) and the MIC was defined as the lowest concentration of peptide that resulted in no apparent growth of the microorganism. From these wells, 10 μl of the overnight culture were added to a Mueller-Hinton agar (MHA) plate and cultured at 37°C for 16–20 h. The lowest concentrations that showed no evidence of colony growth were considered as the MBCs. All the peptide concentrations and controls had 5 replicates in single 96-well plate and three independent experiments were performed.

### Minimum biofilm inhibitory concentration (MBIC) and minimum biofilm eradication concentration (MBEC) assays

MBIC and MBEC assays were performed using microtiter plates based on a modified 2,3,5-triphenyl tetrazolium chloride (TTC) method (Sabaeifard et al., 2014). Overnight cultures were washed with sterile phosphate-buffered saline (PBS) and diluted with fresh broth to 10^7^ CFU/ml. For the MBIC assay, a suspension of inoculum was incubated with the same final peptide concentrations used in the MIC assay (i.e., from 1 to 512 μg/ml) at 37°C for 20–24 h. For the MBEC assay, 200 μl of inoculum was placed in a flat-bottomed microtiter plate for 48 h to form mature biofilms. Following sufficient growth time, mature biofilms were washed twice to remove the planktonic cells and incubated with a series of peptide concentrations (1–512 μg/ml) at 37°C for 20–24 h. After sufficient growth, plates were washed twice with sterile PBS followed by the addition of fresh medium (200 μl per well) and stained with 50 μl 1% TTC (g/v) solution for 5 h. After incubation, 200 μl of the supernatant from each well were transferred to a new plate and their absorbance values were measured at 470 nm using a Synergy HT plate reader (BioTek, USA). Again, all the peptide concentrations and controls had 5 replicates in single 96-well plate and three independent experiments were performed.

### Antimicrobial activities in the presence of serum

The stability of the peptides was investigated by incubating them with serum and evaluating the loss of antimicrobial activity. Twenty-five percent fetal bovine serum/RPMI medium (v/v) that had been pre-equilibrated at 37°C for 15 min was mixed with each peptide to achieve a series of concentrations from 51.2 to 0.1 g/l in two-fold dilutions. Following a 4 h incubation at 37°C, the peptide solutions were briefly centrifuged and 1 μl of each concentration was mixed with 99 μl of diluted bacterial culture (5 × 10^5^ CFU/ml) in a 96-well plate. The plate was then incubated at 37°C for 16–20 h. The MICs in the presence of serum were determined by use of a Synergy HT plate reader (BioTek, USA). Again, all the peptide concentrations and controls had 5 replicates in single 96-well plate and three independent experiments were performed.

### DNA leakage assay

The leakage of DNA and cytoplasmic materials is regarded as an indication of cell membrane lysis (Sahu et al., 2009). As such, we performed a DNA leakage assay to verify the integrity of cell membranes in the presence of AMPs based on the cytoplasmic material-releasing study of Samanta et al. (2013). Briefly, overnight microbial cultures of *S. aureus* and *C. albicans* were collected by centrifugation and the cell pellets were resuspended and washed with pre-warmed PBS and further diluted to 5 × 10^5^ CFU/ml in PBS. Peptide concentrations from 1 to 512 μg/ml were again made in two-fold dilution. PBS and 0.2% Triton-X 100 in PBS were used as negative and positive controls, respectively. All the mixtures were incubated at 37°C for 2 h and then filtered through a 0.22-μm membrane filter. One hundred Microliter of supernatant was transferred into a 96-well plate and analyzed at 260 nm using a plate reader. The DNA leakage percentages of each concentration of peptide solution were then calculated as:

(1) $$\begin{array}{l} \%\text{ DNA leakage }=\frac{A_{x}\;-\;A_{0}}{A\;-\;A_{0}}\;\times \;100\% \end{array}$$

where *A*_*x*_, *A*_0_, and *A* are the optical densities λ260 for the different peptide concentrations, the negative control and the positive control, respectively.

### Haemolysis assay

The haemolysis assay was performed by mixing the peptides with mammalian erythrocytes obtained from fresh defibrinated horse blood. An appropriate volume of horse blood was transferred and washed with PBS until the supernatant was clear. A series of peptide solutions were incubated with a 2% suspension of red blood cells at final concentrations of 1–512 μg/ml at 37°C for 2 h. For positive and negative controls, the red blood cells were incubated with 1% Triton-X 100 and PBS, respectively. After incubation, 200 μl of the supernatant from each sample was transferred to a microtiter plate and the absorbance at 550 nm was measured with a Synergy HT plate reader (BioTek, USA). The therapeutic index (TI), a parameter recommended by the US FDA to indicate safety/efficacy usage, was calculated as the peptide concentration causing 50% haemolysis of the red bloods cells (HC_50_) divided by the geometric mean of the peptide MICs against the relevant bacteria (GM). TI values were calculated for all tested bacterial strains as well as for only those against which the peptide showed an inhibitory effect. In the former case, the MIC that was used when no inhibition was observed was the 2-fold maximum tested value (i.e., 1024 μg/ml) (Irazazabal et al., 2016).

### Peptide design

In this study, the amphipathic α-helical medusin-PT (the natural AMP) was utilized as a template to design a series of peptide analogs. A key change was to introduce additional positive charges to the primary structure to potentially enhance the interaction of the peptide with the negatively-charged microbial membranes and thus potentially modify its biological activity. To do so, we substituted Thr^10^ with lysine to form the analog medusin-PT1. In addition, to investigate the functional stability of optical isomers, a diastereoisomer of medusin-PT1 was designed (using medusin-PT1 as a template) in which L-Leu^2^ was replaced with D-leucine to yield medusin-PT1a (medusin-PT1a). Another analog, medusin-PT2 (medusin-PT2), was designed by replacing Pro^6^ in the natural AMP with lysine to explore the effect of a lengthened helical domain (Loose et al., 2006) given that proline is usually present as the end of a α-helix both because it cannot donate an amide for hydrogen bond formation and because its side chain can result in increased steric hindrance (Thompson et al., 2007). Substitution of Pro^6^, which is part of the nonpolar face of the peptide, with lysine also increased the propensity of the net charge as well as the amphipathicity of the peptide. Both factors should again increase the interaction of the AMP with the microbial cell membrane.

### Molecular modeling, validation of modeled structure, and physicochemical properties of the peptides

Due to the unavailability of an experimental structure template with good identity, the I-TASSER webserver (Yang et al., 2015) was used to predict the secondary structures of the medusin AMPs and to infer the 3D models. Overall qualities of the predicted models were assessed by z-scores using ProSA (Wiederstein and Sippl, 2007) and by Ramachandran plots using RAMPAGE (Lovell et al., 2003). The 3D models of the peptides were rendered with PyMol (The EduPyMOL Molecular Graphics System, Version 1.7.4.5 Schrödinger, LLC). Physiochemical properties of the peptides were predicted by Heliquest (http://heliquest.ipmc.cnrs.fr/) and secondary structures were determined by helical wheel plots obtained from helical wheel projections (http://rzlab.ucr.edu/scripts/wheel/wheel.cgi).

In addition, circular dichroism (CD) analyses were conducted using a JASCO J815 Spectropolarimeter (JASCO Inc., USA) and a quartz cuvette with a 1-mm path length. Each sample was analyzed at 20°C within the range of 190–250 nm. The parameters were set at 100 nm/min scanning speed, a bandwidth of 1 nm, and 0.5 nm data pitch and obtained using three passes (“accumulation”). Peptide samples were dissolved in 10 mM ammonium acetate buffer and 50% TFE in 10 mM ammonium acetate buffer at a concentration of 100 μM. The percentage of the α-helix structure was predicted by the website K2D3 (Louis-Jeune et al., 2012).

### Statistical analyses

Data were analyzed to obtain the mean and standard error of responses. The statistical analyses were performed by one-way ANOVA and the statistical significance of the differences was indicated as ^*^p < 0.05, ^**^p < 0.01, ^***^p < 0.001, and ^****^p < 0.0001. The dose-response curves were constructed using a “best-fit” algorithm and HC_50_ was calculated through the data analysis package provided in GraphPad Prism 6 (GraphPad Software, USA).

## Results

### Molecular cloning of a novel AMP precursor-encoding cDNA from the library constructed from skin secretion and prediction of the primary structure of the novel AMP

**Figure 1:**
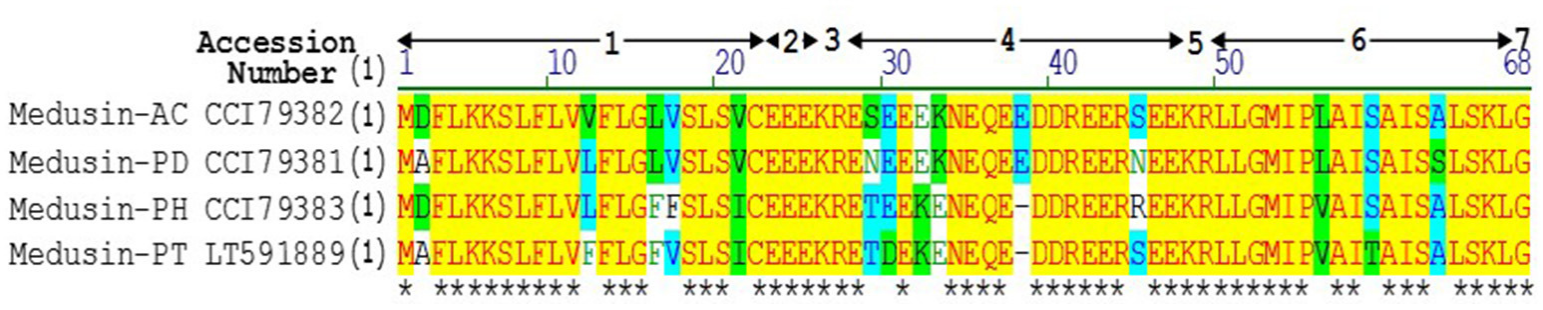
**Sequence alignment of precursors of medusin-AC, medusin-PD, medusin-PH, and medusin-PT**. 1. Putative signal peptide. 2. Acidic spacer peptide region. 3. Propeptide convertase processing site. 4. Acidic spacer peptide region. 5. Propeptide convertase processing site. 6. Mature peptide. 7. Glycine residue amide donor. Conserved residues are indicated in yellow highlight and asterisks. Adapted from sequences published in Xi et al. (2013).

**Figure 2:**
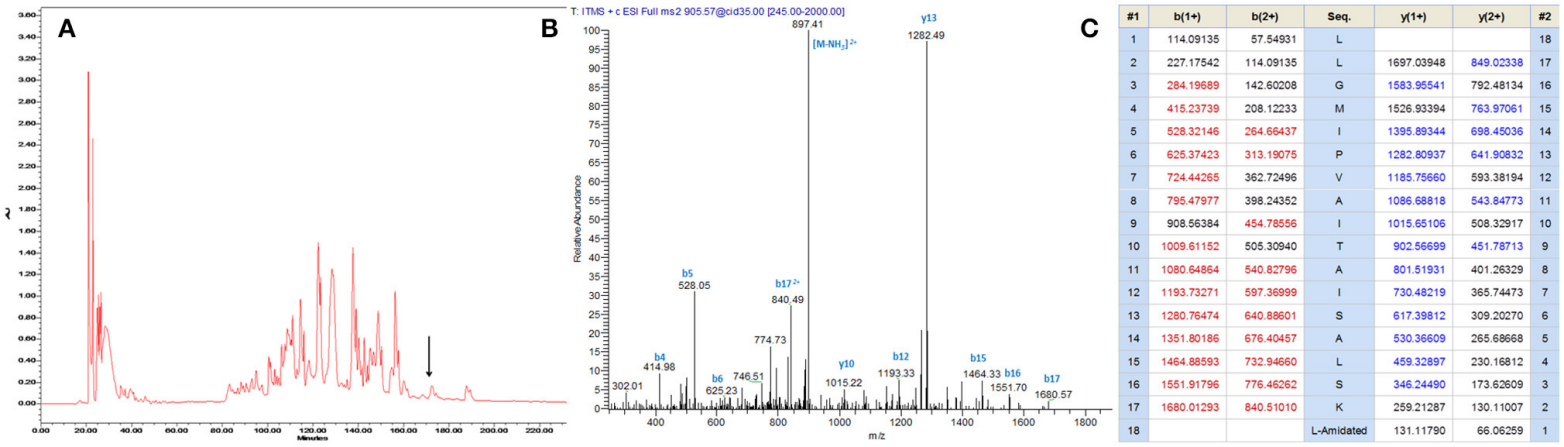
**(A)** Reverse phase HPLC chromatogram of skin secretion of *P. tarsius* monitored at 214 nm. The arrow indicates the retention time of medusin-PT. **(B)** Annotated fragment ion spectrum of medusin-PT. **(C)** Predicted *b-* and *y-*ions arising from collision induced dissociation (CID) of the doubly-charged (905.57 m/z, [M+2H]^2+^) precursor ion. The observed *b-* and *y-*ions are indicated in blue and red typefaces.

A single transcript was repeatedly cloned and its nucleic acid and translated amino acid sequences were determined (Supplementary Figure 1). The open-reading frame consisted of 67 amino acids and contains the classic Lys-Arg (KR) processing site so as to derive the mature peptide (Figure 1). The fractions of skin secretion resolved by HLPC (Figure 2A) that yielded the predicted peptide mass were further analyzed by MS/MS fragmentation (Figures 2B,C). The *y-*ions of medusin-PT were found to be 1 Da less than the calculated value, confirming the amidation modification of the C-terminus.

### Peptide design

**Figure 3:**
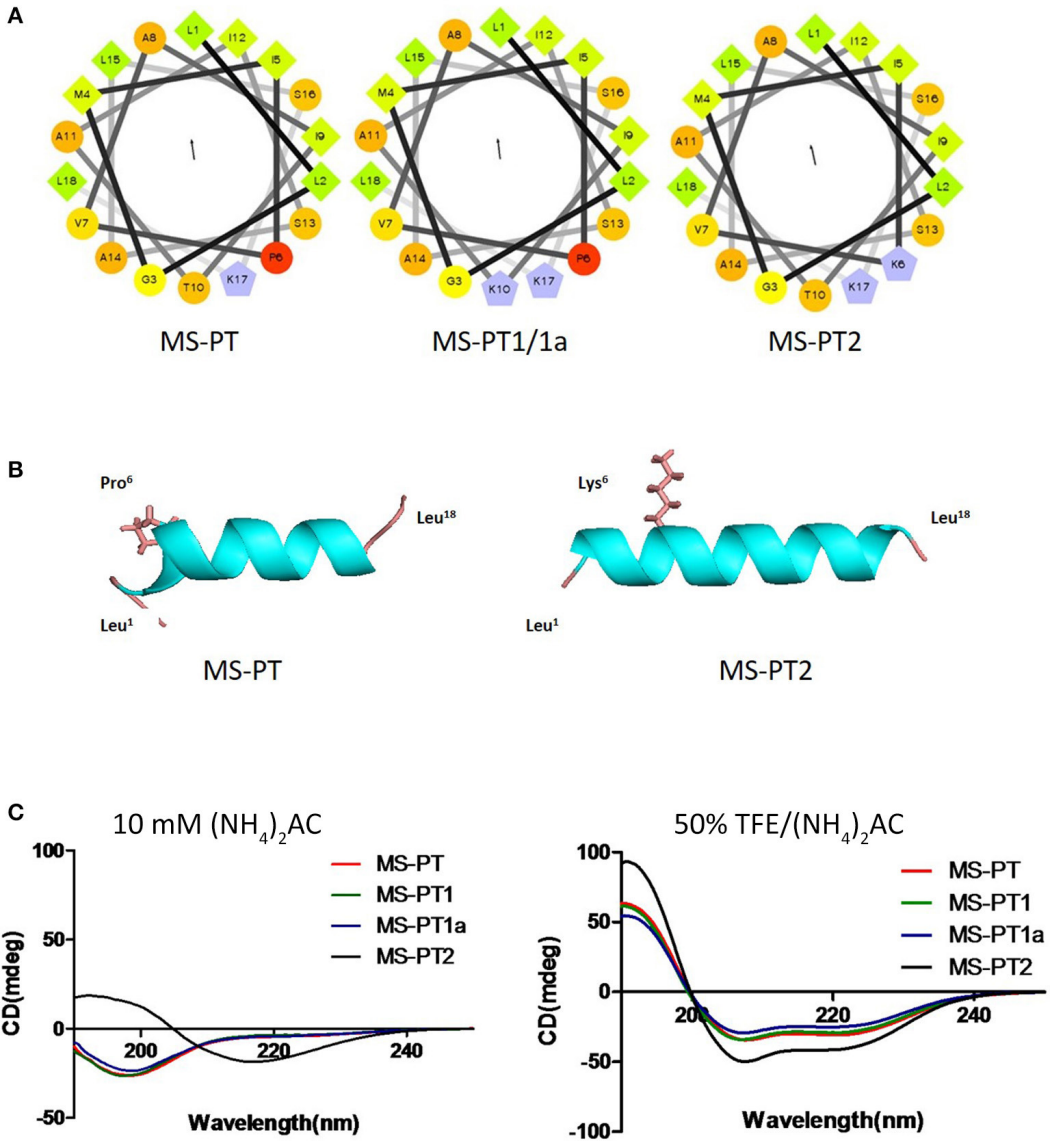
**(A)** Helical wheel projections of the four peptides medusin-PT, medusin-PT1, medusin-PT1a, and medusin-PT2, with arrows indicating the direction of summed vectors of hydrophobicity. **(B)** Predicted 3D models of medusin-PT and medusin-PT2 rendered with PyMol. Side chains of the modified position 6 were shown and other side chains were neglected. The modified peptide Medusin-PT2 showed a clearly enhanced helical structure compared with the native peptide medusin-PT. **(C)** Superimposition of CD spectra recorded for the four medusin peptides (100 μM) in 10 mM in ammonium acetate buffer and in 50% TFE ammonium acetate buffer.

Based on the natural peptide, we designed three analogs emphasizing the effects of an increased positive charge, an elongated α-helical domain and D-amino substitutions (Figure 3A and Table 1). Both z-scores and Ramachandran plots indicated good structural qualities for the models of medusin-PT and medusin-PT2 predicted by I-TASSER. z-scores of both peptides (Figure 3B) were within the range of scores typically found for peptide native folds of similar size (Supplementary Figure 2). Furthermore, the stereo-chemical backbone characters of the two models were evaluated by analyzing backbone and Psi dihedral angles (Ramachandran plot; Supplementary Figure 3) and found to be of good quality and reliable for downstream analysis. According to the predicted data, our directed modifications resulted in an increase in both the hydrophobic moment and the overall charge of each modified peptide compared to the natural medusin-PT. Prediction of the peptide secondary structure by CD (Figure 3C) revealed typical α-helical bands in the CD spectra at around 207 and 222 nm in 50% TFE ammonium acetate buffer. An unordered conformation was detected only in 10 mM ammonium acetate buffer. Compared with the results from the natural peptide, the intensity of the bands at 207 and 222 nm for medusin-PT2 were significantly augmented. Consistent with our expectation, the α-helical propensity was also simultaneously generally increased, although the helicity of medusin-PT1a was slightly diminished compared to that of medusin-PS1, possibly because the D-amino acid substitution introduced a kink in the regular helical structure. The α-helical content of medusin-PT2 was the highest among the peptides at about 70%. The α-helical propensities of the other peptides were similar, at around 50% (Table 1).

**Table 1:** **The predicted physicochemical parameters and calculated percentage of helical content of medusin-PT, medusin-PT1, medusin-PT1a, and medusin-PT2**.

**Peptide**	**Sequence**	**Hydrophobicity (H)**	**Hydrophobic moment (μH)**	**Net charge**	**% helix**	
					**10 mM ammonium acetate**	**50% TFE**
medusin-PT	LLGMIPVAITAISALSKL-NH_2_	0.861	0.335	2	7	53
medusin-PT1	LLGMIPVAIKAISALSKL-NH_2_	0.791	0.404	3	6	52
medusin-PT1a	LlGMIPVAIKAISALSKL-NH_2_	0.791	0.404	3	7	43
medusin-PT2	LLGMIKVAITAISALSKL-NH_2_	0.766	0.414	3	26	70

*D-type amino acid substitution was indicated with lowercase letter*.

### Bioactivity assays

**Figure 4:**
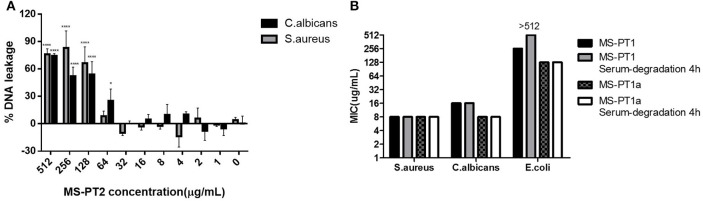
**(A)** Minimum inhibitory concentrations (MICs) of medusin-PT, medusin-PT1, medusin-PT1a, and medusin-PT2 with or without serum degradation against *S. aureus, E. coli*, and *C. albicans*. **(B)** Effect of medusin-PT2 on DNA leakage recorded at 260 nm. *S. aureus* (gray) and *C. albicans* (black) are indicated. The error bar represents the standard error for three repeats. ^*^p < 0.05, ^**^p < 0.01, ^***^p < 0.001, ^****^p < 0.0001 significant difference between the absorption of test peptide concentration and the absorption of negative control (no peptide).

The natural peptide and its analogs exhibited different degrees of inhibition against the growth of the tested microorganisms (Table 2). Compared to the modified peptides, the natural AMP medusin-PT possessed comparatively weak bioactivity and only against *S. aureus* (MIC of 64 μg/ml). By contrast, the designed peptides showed enhanced bioactivity. All were potent against *S. aureus* as well as *C. albicans*, with the inhibition against these two microorganisms being increased 8-fold. DNA leakage experiments for the analog medusin-PT2 against the Gram-positive bacterium *S. aureus* and the yeast *C. albicans* revealed that leakage was only observed at peptide concentrations higher than the respective MIC and MBC values for these two microbes (Figure 4A). The three modified peptides were also potent against *E. faecalis* (MIC of 32 μg/ml). No obvious inhibitory effect was observed against the Gram-negative *E. coli* and *P. aeruginosa* by either medusin-PT or medusin-PT2. Indisputably, medusin-PT1 and medusin-PT1a were the most effective peptides, being highly inhibitory against *S. aureus* (MIC of 8 μg/ml), but also moderately inhibitory against *E. coli* (MIC and MBC of 128 μg/ml). Finally, all the modified peptides displayed potent bacteriostatic and bactericidal activity against MRSA, inhibiting the growth of this bacterium at a range of concentrations from 16 to 32 μg/ml.

**Table 2:** **Minimum inhibitory concentrations (MICs), minimum bactericidal concentrations (MBCs), and therapeutic indices (TI) of medusin-PT, medusin-PT1, medusin-PT1a, and medusin-PT2 against reference microorganisms**.

**Strains**	**MIC/MBC (**μ**g/ml)**			
	**medusin-PT**	**medusin-PT1**	**medusin-PT1a**	**medusin-PT2**
*S. aureus*	64/128	8/16	8/16	8/16
MRSA	>512/>512	16/32	16/32	32/32
*E. faecalis*	>512/>512	32/32	32/32	32/32
*C. albicans*	>512/>512	16/16	8/8	8/8
*E. coli*	>512/>512	256/512	128/128	>512>512
*P. aeruginosa*	>512/>512	>512/>512	128/>512	>512/>512
HC_50_	1.814 × 10^6^	102.2	226.7	93.83
TI (overall)	2812.06	2.01	7.95	1.47
TI (Gram-positive and yeast)	3542.97	6.39	16.85	5.86

Similarly, the natural peptide medusin-PT could only slightly inhibit the formation of biofilm by *S. aureus* (MBIC of only 512 μg/ml; Table 3). By contrast, all of medusin-PT1, medusin-PT1a, and medusin-PT2 were able to maintain potent inhibition of the sessile bacterial cells (MBIC of 32 μg/ml) together with significant increases in MBEC values compared to the natural peptide (128, 64, and 256 μg/ml, respectively).

**Table 3:** **The biofilm formation inhibition and biofilm eradication activity of medusin and the analog against ***S. aureus*** biofilm**.

	**MBIC (μg/ml)**	**MBEC (μg/ml)**
medusin-PT	512	>512
medusin-PT1	32	128
medusin-PT1a	32	64
medusin-PT2	32	256

In serum conditions (Figure 4B), both medusin-PT1 and medusin-PT1a exhibited steady antimicrobial activities against both *S. aureus* and the yeast *C. albicans*, and thus no obvious functional changes compared to the standard assays. However, both peptides showed different behaviors against *E. coli*: medusin-PT1 completely lost its inhibitory activity after incubation with serum for 4 h whereas medusin-PT1a maintained its potency.

Finally, whereas medusin-PT showed an extremely low haemolytic activity, the designed peptides induced a significant increase in haemolysis of the horse erythrocytes (Table 2), thereby causing medusin-PT to possess the highest TI values despite not having any detectable antimicrobial activities for the most part. Importantly, however, the three modified peptide analogs had enhanced antimicrobial activities, but still maintained acceptable TI values.

## Discussion

Based on its primary structure, the newly isolated peptide medusin-PT (as well as medusin-AC, medusin-PD, and medusin-PH) clearly belongs to the medusin AMP family, a recently discovered AMP family in the skin secretions of phyllomedusine frogs (Xi et al., 2013). Like other medusins (Figure 1), it is of a similar length (ca. 18 amino acids) and hydrophobicity (ca. 70%) and contains a single lysine residue and C-terminal amidation through post-translational modification of the C-terminal glycine residue, which acts as an amide donor. This modification also increases the cationic charge. Moreover, it also shares the same conserved signal peptide sequence as well as the signature patterns of an N-terminal hexapeptide sequence (LLGMIP-), a consistent internal sequence (-AISAIS-), and a C-terminal tetrapeptide amide sequence (-LSKL-NH_2_) (Xi et al., 2013). The bioactivity of medusin-PT also matched that of other medusins in being effective against only planktonic *S. aureus* and with low haemolytic activity. In addition, there was no bioactivity against biofilms, even those formed by *S. aureus*.

Although medusin-PT therefore qualifies as a possible drug candidate, especially given its high TI values, the example of Pexigana, which is modified from magainin and has been shown to be useful to treat infection related diabetic foot ulcers in clinical trials (Hancock and Sahl, 2006), opens the possibility that directed modifications of this natural AMP might improve its bioactivity against a broader range of pathogens, while maintaining or even lowering its cytoxicity. In this regard, key parameters for such a template-designed approach include overall net charge, α-helicity, hydrophobicity, and amphipathicity (Fjell et al., 2012). We focussed primarily on altering the net charge of the peptide (in medusin-PT1), but also with a contribution from increasing its α-helicity (medusin-PT2). The first two of these properties in our designed analogs. Both peptide analogs showed generally increased bioactivity, both against *S. aureus* (also as biofilms) as well as against most of the other tested organisms, including MRSA. This increased efficacy and broader action came at the cost of increased haemolytic activity as well, thereby dropping the TI values by three orders of magnitude compared to the natural form of the peptide. Fortunately, however, the HC_50_ values for the analogs remained lower than most of the MIC values indicating their possible therapeutic usage as a broad-spectrum antibiotic.

Another consideration for the use of AMPs as drug candidates is their susceptibility to break down within the patient (e.g., via serum degradation). To guard against this, another design concept is to replace the natural amino acids by their D-amino acids isomers at potential cleavage sites. In creating medusin-PT1a, the diastereoisomer of medusin-PT1, we targeted Leu^2^ because the position was far enough away from the α-helical core to minimize any potential disruption of the α-helical structure. This change showed numerous positive effects in that bioactivity compared to medusin-PT1 was maintained, if not occasionally improved and cytotoxicity was reduced so as to yield the highest TI value among the designed analogs. Moreover, medusin-PT1a retained its activity against the Gram-negative *E. coli* after serum incubation whereas it was lost in medusin-PT1. From this result, we infer that the degraded fragments of the AMPs following serum degradation remain long enough to disrupt the cell membranes of *S. aureus* and *C. albicans*, but not to interact with that of *E. coli*.

As α-helical cationic peptides, medusins are considered to interact with and permeabilise the lipid bilayer of bacterial membranes. However, from the different activities of the medusins against Gram-positive and Gram-negative bacteria, it is clear that differences in the structures of outer membranes can induce differential susceptibilities (Dȩbowski et al., 2012). In particular, the outer membrane of Gram-negative bacteria could trap the AMPs to prevent their further translocation thereby protecting the cytoplasmic membrane. Another hypothesis holds that the extent of initial interaction with the cell membrane could also be modulated by cell-wall components such as peptidoglycan, which can either entrap AMPs or promote their accumulation on the cytoplasmic membrane (Melo et al., 2009). Interestingly, the DNA leakage assay indicated that the antimicrobial actions of at least medusin-PT2 might have different patterns depending on the AMP concentration, with a non-membrane permeabilisation mode apparently being used at MIC-like concentrations, but with significant disruption of the membrane integrity at higher concentrations.

Likewise, biofilms, as extracellular matrix structures of certain bacteria, act to shield the bacterial cells from the hazards of the external environment. In particular, sessile bacteria could experience more than 100-fold protection against antimicrobial agents compared to the planktonic bacteria that are constantly released from the biofilm (Costerton, 1999; Harisson, 2006). Although all the medusin AMPs we examined were able to inhibit the growth of the biofilm of *S. aureus*, their bioactivities were much reduced compared to those against planktonic *S. aureus* and only present at effective levels for the medusin analogs we synthesized. Importantly, the medusin-PT1a was more effective than its diastereoisomer medusin-PT1, indicating that protease-resistant peptides might perform more effectively against biofilms. Although the mechanism of action of AMPs against biofilms remains unknown, it might be that the AMPs are transferred through the extracellular biofilms via holes or pores formed in lipid component of this layer or just disperse the biofilms outright (Park et al., 2011).

In conclusion, we have demonstrated that directed modifications of a natural AMP can indeed increase its bioactivity, albeit with a concurrent, but smaller increase in cytotoxicity in this particular case. In our study, it is difficult to disentangle the relative importance of increasing the net charge versus the α-helicity with respect to improving the bioactivity of medusin-PT. Although increasing the α-helicity via the Pro to Lys substitution had only minor effects (compare medusin-PT1 vs. medusin-PT2), it has been reported that a central proline residue might also be important for biological activity by any or all of enhancing translocation promoting factors, influencing the folding of bacteriorhodopsin or aiding the folding of membrane peptides (Cordes et al., 2002). In conjunction with the last point, we note that the medusin peptides underwent a coil-to-helix transition in going from a water environment to a membrane mimic environment, indicating that a structural transformation might take place when they interact with bacterial membranes. However, increasing the net positive charge has also found to be helpful in other studies (Gao et al., 2016; Wu et al., 2016), presumably by increasing the initial electrostatic attraction between the medusin AMP and the bacteria cell membrane, which otherwise presents a major hindrance for antimicrobial actions. In line with the observation, that our modifications increased the bioactivity of medusin-PT against the superbug MRSA, this study demonstrates that, through a rational design method, it is possible to design peptide-based therapeutics that are active against a broad range of microorganisms including those that are resistant to conventional drugs.

## Author contributions

The conception and design of study was conducted by LW, MZ, and TC. The laboratory work and acquisition of data was performed by YG, DW, and CM. The analysis of data was conducted by LW, CL, XX, and JD. The manuscript was drafted by YG, DW, and MZ and written and revised by OB, XX, TC, and CS.

### Conflict of interest statement

The authors declare that the research was conducted in the absence of any commercial or financial relationships that could be construed as a potential conflict of interest.
