# Supplementary material for: Targeted Modification of a Novel Amphibian Antimicrobial Peptide from Phyllomedusa tarsius to Enhance Its Activity against MRSA and Microbial Biofilm
Source: Front Microbiol. 2017 Apr 19;8:628. doi: 10.3389/fmicb.2017.00628 (PMC5395648; doi:10.3389/fmicb.2017.00628)
Supplement: Supplementary file 1 [file DataSheet1.docx]

Supplementary Material

Targeted modification of a novel amphibian antimicrobial peptide from Phyllomedusa tarsius to enhance its activity against MRSA and microbial biofilm

Yitian Gao ^1, *^, Di Wu^1, *^, Lei Wang^1^, Chen Lin^1,2^, Chengbang Ma^1^, Xinping Xi^1, **^, Mei Zhou^1, **^, Jinao Duan^3^, Olaf R. P. Bininda-Emonds^4^, Tianbao Chen^1^ and Chris Shaw^1^

*** Correspondence:** These authors contributed equally to this work.

^**^ Corresponding author. Fax: ++44 28 90247794

E-mail: [x.xi@qub.ac.uk](mailto:x.xi@qub.ac.uk) (X. Xi); [m.zhou@qub.ac.uk](mailto:m.zhou@qub.ac.uk) (M. Zhou)

## Supplementary Figures


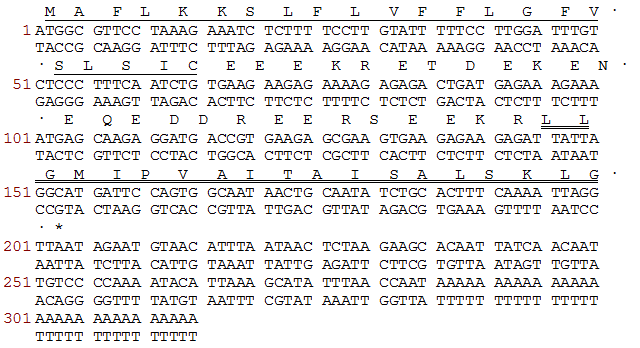


**Supplementary Figure 1.** Nucleotide and translated open-reading frame amino acid sequences of the cloned cDNA encoding the biosynthetic precursor of Medusin-PT. The putative signal peptide is single-underlined, the mature peptide is double-underlined and the stop codon is indicated by an asterisk.


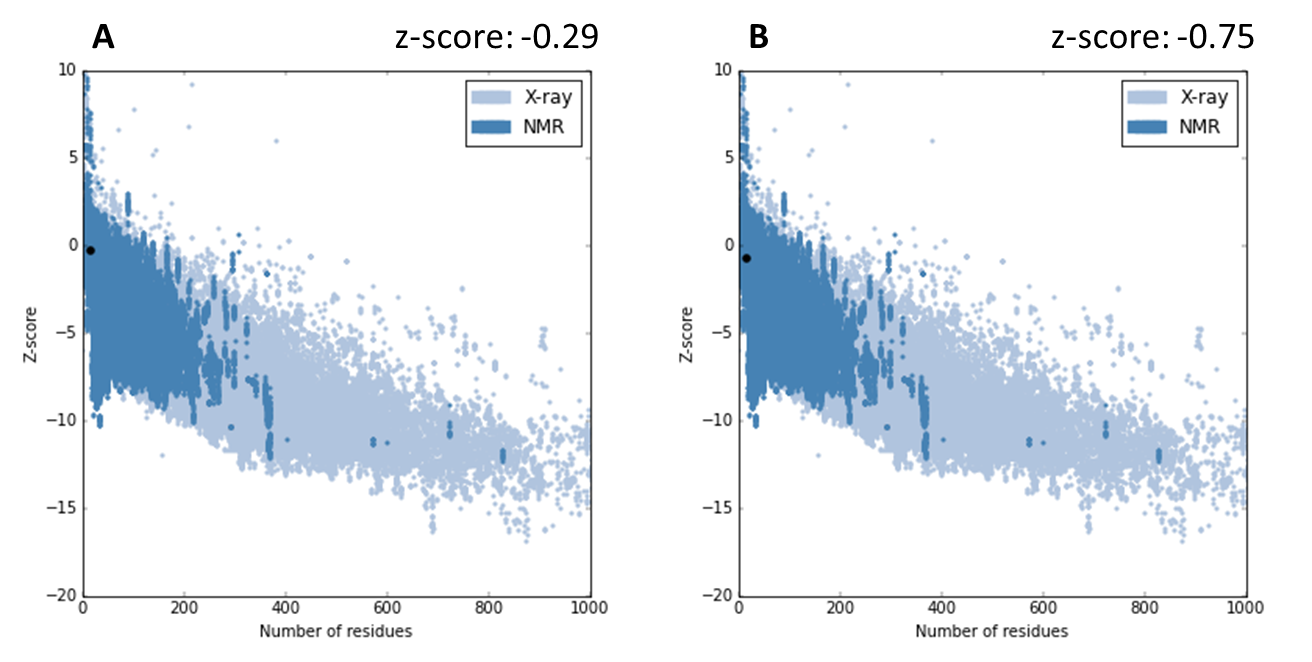


**Supplementary Figure 2.** ProSA model assessment for medusin-PT (A) and medusin-PT2 (B), z-scores of the two peptide models were given and were within the range of scores of all experimentally determined (X-ray, NMR) protein structures in PDB.


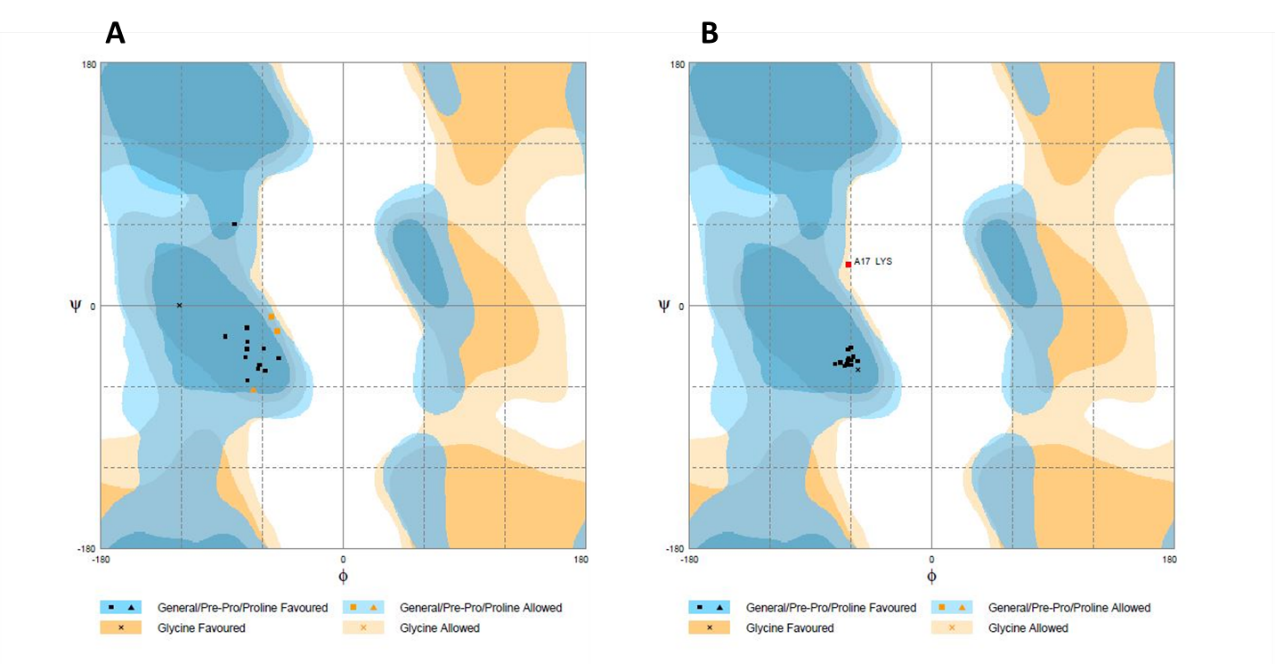


**Supplementary Figure 3.** Ramachandran plot of the 3D models of medusin-PT (A) and medusin-PT2 (B). All residues of medusin-PT were in favoured and allowed regions, while for medusin-PT2, only the K17 residue was in the outlier regions (but near the allowed region) and all the other residues were in favoured region.
